# Identification and Mechanism of *Echinochloa crus-galli* Resistance to Fenoxaprop-p-ethyl with respect to Physiological and Anatomical Differences

**DOI:** 10.1100/2012/893204

**Published:** 2012-04-19

**Authors:** Amany Hamza, Aly Derbalah, Mohamed El-Nady

**Affiliations:** ^1^Pesticides Department, Faculty of Agriculture, Kafrelshiekh University, Kafr el-Sheikh 33516, Egypt; ^2^Department of Agricultural Botany, Faculty of Agriculture, Kafrelshiekh University, Kafr el-Sheikh 33516, Egypt; ^3^Department of Biology, Faculty of Applied Science, Taibah University, P.O. Box 344, Al Madinah Al-Munawwarah, Saudi Arabia

## Abstract

Identification and mechanism of *Echinochloa crus-galli* (L.) resistance to fenoxaprop-p-ethyl via physiological and anatomical differences between susceptible and resistant were investigated. The physiological and anatomical differences that were take into account were growth reduction, chlorophyll content reduction, lamina thickness, and xylem vessel diameter in both susceptible and resistant biotypes of *E. crus-galli*. The results showed that the growth reduction fifty (GR_50_) of resistant biotype was 12.07-times higher than that of the susceptible biotype of *E. crus-galli* treated with fenoxaprop-p-ethyl. The chlorophyll content was highly reduced in the susceptible biotype relative to the resistant one of *E. crus-galli* treated with fenoxaprop-p-ethyl. An anatomical test showed significant differences in the cytology of susceptible and resistant biotypes of *E. crus-galli* treated with fenoxaprop-p-ethyl with respect to lamina thickness and xylem vessel diameter. The resistance of *E. crus-galli* to fenoxaprop-p-ethyl may be due to the faster metabolism of fenoxaprop-p-ethyl below the physiologically active concentration or the insensitivity of its target enzyme (Acetyl-CoA carboxylase).

## 1. Introduction

*E. crus-galli* (L.)* Beauv* is a type of wild grass originating from tropical Asia that was formerly classified as a type of panicum grass. Considered as one of the world's worst weeds, it reduces crop yields and causes forage crops to fail by removing up to 80% of the available soil nitrogen. The high levels of nitrates it accumulates can poison livestock. It acts as a host for several mosaic virus diseases. Heavy infestations can interfere with mechanical harvesting. Individual plants can produce up to 40,000 seeds per year. Water, birds, insects, machinery, and animal feet disperse it, but contaminated seed is probably the most common dispersal method. More than 35% of grain yield in seeded rice was reduced by infestation with *E. crus-galli* [1].

Due to the great risk of this weed infestation in Egypt and worldwide, herbicide is becoming the most popular method of weed control in rice. However, while herbicide application certainly controls the weeds, experience shows, however, that although herbicide use alleviates the problem of labor for weeding, incorrect use of herbicides may bring about other environmental problems such as selecting for resistance to herbicides. Weed resistance to herbicides concerns many sectors of the agricultural community: farmers, advisors, researchers, and the agrochemical industry in Egypt and worldwide. The fear exists that in an extreme case of resistance, farmers might lose a valuable chemical tool that had previously provided effective control of yield-reducing weeds. Resistance is often seen as a problem caused by a particular active ingredient. This is an over-simplification and a misconception. Resistance results from agronomic systems which have been developed to rely too heavily on herbicides as the sole method of weed control [2]. Without monitoring and rapid detection of the resistance evolution, interpretation of its mechanism and trying to find sustainable management strategies, the future usefulness of herbicides as a tool for weed control might be seriously jeopardized. Furthermore, the identification of resistance mechanism to herbicides is considered the key step toward developing appropriate solutions to overcome this phenomenon.

Resistance mechanism through evaluation of the activity of target site enzymes has been reported before [3–5]; however, characterizing the resistance mechanisms of weeds to herbicides via investigating the anatomical and physiological differences in susceptible and resistant biotypes considered a source of major concern and has not been studied before.

Therefore, this study attempted to identify the occurrence and mechanism of *E. crus-galli*-resistant biotype against fenoxaprop-p-ethyl via investigation of the physiological (chlorophyll content and growth reduction) and anatomical differences between the susceptible and resistant biotypes of *E. crus-galli *(barnyardgrass) treated with fenoxaprop-p-ethyl.

## 2. Materials and Methods

### 2.1. The Used Herbicide

Fenoxaprop-p-ethyl with the trade name of Whip-super EW 7.5% was obtained from Rice Weeds Research Department, Rice Research and Training Center, Sakah, and Kafr el-Sheikh, Egypt. This herbicide was applied at the filed rate of 49.5 gm a.i/hectare.

### 2.2. The Tested Weed

The susceptible biotype (SBT) of the *Echinochloa crus-galli* to fenoxaprop-p-ethyl (obtained from Rice Weeds Research Department, Rice Research and Training Centre, Sakah, Kafr El-Sheikh). The resistant biotype (RBT) of *Echinochloa crus-galli* used in this study was previously treated for several years with the tested herbicide by selection pressure and recorded resistance [6].

### 2.3. Whole Plant Bioassay

Dose-response experiments were conducted at the greenhouse of the Agricultural Botany Department, Faculty of Agriculture, Kafr El-Sheikh University, Egypt. The soil used in this experiment was fertilized with nitrogen at a rate of 360 kg/h of urea fertilizer (containing 46% nitrogen). Super phosphate fertilizer (phosphorus 15%) was added at a rate of 240 kg/ha before planting. Potassium was not added because the Egyptian soil is rich in this element. Seeds of susceptible and resistant biotypes of *Echinochloa crus-galli* were planted in 30 × 30 cm plastic pots filled with soil. Emerged seedlings were thinned to four uniform and equally distant-spaced plants per pot. These experiments were conducted at average daily temperatures ranging from 22 to 31°C and at a 16-h day length. Pots were immersed with water up to 4 cm above the soil surface. The tested herbicide, fenoxaprop-p-ethyl, was applied as a single application using a hand sprayer at the 4-leaf to 1-tiller stage of growth of the tested weed. The concentration levels used were 0.1, 0.5, 1, and 2 folds of fenoxaprop-p-ethyl recommended dose. After forty-eight hours of treatment, the plants were irrigated and water was raised up to 4 cm above the soil surface [4]. Experiments were done in a completely randomized design with six replicates. Fresh weight of treated and untreated plant was determined after 14 days of fenoxaprop-p-ethyl application. Data were pooled and fitted to a log-logistic regression model [7, 8] as shown in  (1)

(1) $$Y=c+\left\{\frac{\left(d-c\right)}{\left[1+{\left(x/g\right)}^{b}\right]}\right\},$$

where *Y* is the fresh weight of germinated seedling aboveground expressed as percentage of the untreated control, *c* and *d* are the coefficients corresponding to the lower and upper asymptotes, *b* is the slope of the line, *g* (GR_50_) is the herbicide rate at the point of inflection halfway between the upper (*d*) and lower (*c*) asymptotes, and *x* (independent variable) is the herbicide dose.

Regression analysis was conducted using the Sigma Plot statistical software version 10.0 [4]. The herbicide rate used to reduce plant growth by 50% relative to the untreated control (GR_50_) was calculated for resistant and susceptible biotypes of* E. crus-galli*. R/S ratios were calculated as the GR_50_ of the resistant (R) biotype divided by the GR_50_ of the susceptible (S) biotype.

### 2.4. Chlorophyll Measurements

Plant leaves differ from that used in fresh weight determination were used to determine the chlorophyll content of resistant and sensitive biotypes of* E. crus-galli* after 14 days of treatment with fenoxaprop-p-ethyl at the level applied in the real field conditions. Chlorophyll content of untreated controls was measured after 14 days also. Moreover, chlorophyll content of resistant treated and untreated biotypes were remeasured after 21 days of treatment with fenoxaprop-p-ethyl (treated leaves were regrown again and chlorophyll content increased). No chlorophyll data was taken for the suscipble biotype after 21 days due to the plant is completely dead. Chlorophyll A, B, and total were determined in *E. crus-galli* lamina using the method described by Moran and Porath [9]. Data were subjected to statistical analysis of variance according to the method described by K. A. Gomez and A. A. Gomez [10].

### 2.5. Anatomical Test

The leaf specimens which included the midrib collected from plants differ from that used in fresh weight and chlorophyll determination were taken after 14 days of treatment from the second leaf of the resistant and susceptible biotypes of *E. crus-galli* treated with fenoxaprop-p-ethyl at the recommended dose level (1 fold). Moreover, leaf specimens of resistant treated and untreated biotypes were measured again after 21 days of treatment with fenoxaprop-p-ethyl (treated leaves were recovered again and chlorophyll content increased). No leaf specimens were taken for the susceptible biotype after 21 days due to the plant is completely dead. Specimens were fixed in a formalin, ethyl alcohol, and acetic acid mixture (1 : 18 : 1 v/v). Then specimens were washed and dehydrated in an alcohol series. The dehydrated specimens were infiltrated and embedded in paraffin wax (52–54°C m.p.). The embedded specimens were sectioned using a rotary microtome (Leica RM 2125) to a thickness of 8–10 *μ*m. Sections were mounted on slides and deparaffinized. Staining was accomplished with safranine and azur II [11], cleared in xylol, and mounted in Canada balsam [12]. Ten readings from 3 slides from different leaves of the same plant were examined with electric microscope (Lieca DM LS) with digital camera (Lieca DC 300) and then photographed. The anatomical manifestation was calculated using Lieca IM 1000 image manager software. Lieca software was calibrated using 1 cm stage micrometer scaled at 100 *μ*m increment (Leitz Wetzler, Germany 604364) at a 4 and 10x magnifications.

### 2.6. Statistical Analysis

Data from the experiments were statistically analyzed using one-way repeated measurement analysis of variance according to the method described by K. A. Gomez and A. A. Gomez [10]. Duncan's multiple range test was used to separate means using SAS software (version 6.12, SAS Institute Inc., and Cary, USA).

## 3. Results

### 3.1. *E. crus-galli* Resistance to Fenoxaprop-p-ethyl by Means of Fresh Weight Reduction of Treated Plants

A dose-response experiment was conducted on whole plants of *E. crus-galli* treated with fenoxaprop-p-ethyl to detect its resistance against this herbicide. The response of the tested susceptible and resistant biotypes against this herbicide was determined as reduction in the fresh weight of the treated plants relative to the control after 14 days of fenoxaprop-p-ethyl application. The results showed that the rates of fenoxaprop-p-ethyl required for 50% growth reduction were 3 and 36.2 gm a.i./ha for the susceptible and resistant biotypes of* E. crus-galli*, respectively (Table 1). Table 1 revealed that the GR_50_ of *E. crus-galli*-resistant biotype was 12.07-times higher than that required to obtain the same effect on the susceptible biotype.

### 3.2. Effect of Tested Herbicide on Chlorophyll Content of Susceptible and Resistant Biotypes of *E. crus-galli*

The chlorophyll content of *E. crus-galli* was measured after 14 days of herbicide application to evaluate the physiological conditions of the tested weed.  Table 2 showed that the chlorophyll content after fenoxaprop-p-ethyl application was decreased either in the resistant or susceptible biotypes. The rate of reduction in chlorophyll content was higher in susceptible biotype than the resistant one of *E. crus-galli*. The chlorophyll content was higher in the untreated susceptible biotype of *E. crus-galli* relative to the treated one. The chlorophyll content was slightly higher in the untreated resistant biotype of *E. crus-galli* relative to the treated one. A very important action took place. Chlorophyll content of the resistant biotype treated with fenoxaprop-p-ethyl increased again relative to the untreated resistant plants after 21 days of treatment. This action was due to the re-growth of *E. crus-galli* leaves as shown in Table 3.

### 3.3. Anatomical Differences between Susceptible and Resistant Biotypes of *E. crus-galli* against Fenoxaprop-p-ethyl

**Figure 1:**
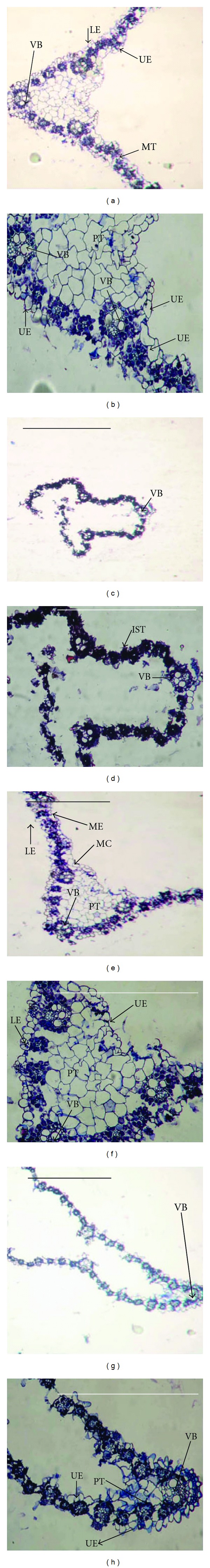
Cross-sections through the lamina of untreated susceptible biotype (a, b), treated susceptible biotype (c, d), untreated resistant biotype (e, f), and resistant biotype treated with fenoxaprop-p-ethyl (g, h) of *E. crus-galli *after 14 days. *Upper epidermis (UE), lower epidermis (LE), parenchyma tissue (PT), mesophyll tissue (MT), motor cells (MC), vascular bundle (VB), and intensive stained tissue (IST); Bar = 500 *μ*m.

The anatomical differences between the susceptible and resistant biotypes of *E. crus-galli *treated with fenoxaprop-p-ethyl with respect to lamina thickness and xylem vessel diameter are presented in Table 4 and Figure 1. The results showed that susceptible biotype (SBT) treated with fenoxaprop-p-ethyl had less laminal thickness and tissues intensively stained with azur II compared with the untreated plants. The normal internal leaf structure of treated SBT is more difficult to be identified, which may be due to cell death compared with untreated plants. Laminal thickness and xylem vessel diameter of treated biotypes were reduced compared with the untreated plants, but the lowest value was caused by treated SBT. In contrast, leaf tissues in treated resistant biotype (RBT) seem to be normal and easily identified. Intensively stained cells with azur II, though, were noticed in some local lesions (areas), which may be due to cell death.

**Figure 2:**
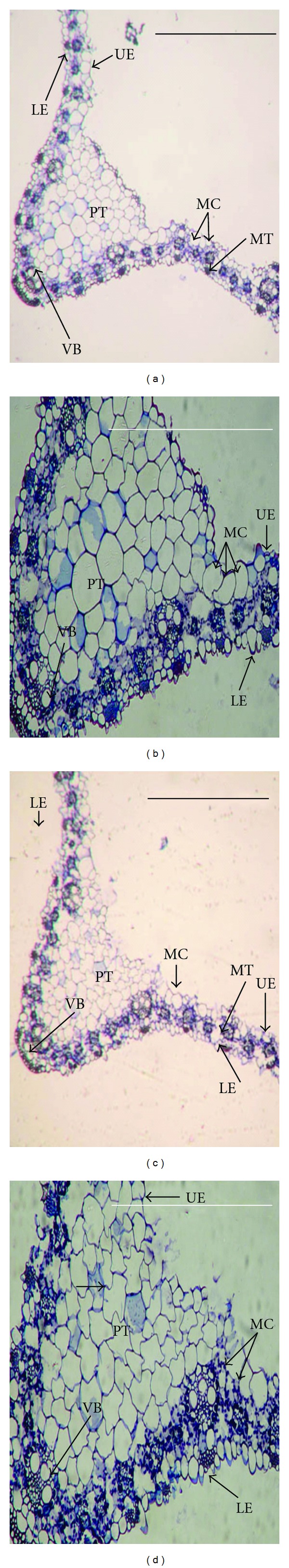
Cross sections through the lamina of untreated resistant (a, b) and treated resistant with fenoxaprop-p-ethyl biotypes (c, d) of *E. crus-galli* after 21 days.

Concerning of recovery in RBT of *E. crus-galli* treated with fenoxaprop-p-ethyl, relative to the untreated one, data in Table 5 and Figure 2 indicated that lamina thickness was increased up to untreated RBT. Furthermore, no differences in xylem vessel diameter were found between treated and untreated RBT.

## 4. Discussion

The resistance of* E. crus-galli* to fenoxaprop-p-ethyl (ACCase inhibitor) was identified in this study and confirmed the occurrence of* E. crus-galli* resistance to fenoxaprop-p-ethyl in Egypt. This finding had been reported previously outside Egypt [3, 13–17]. The results of this study also implied that the physiological and anatomical dereferences as well as growth reduction help to identify the occurrence of resistant weed.

Chlorophyll content has been known as a typical parameter for evaluating the physiological conditions in common sense. The reduction in chlorophyll content of *E. crus-galli *resistant and susceptible biotypes after foliar application of fenoxaprop-p-ethyl in this study is in agreement with the findings of [18, 19] who reported that the application of fenoxaprop-p-ethyl as ACCase-inhibitor leads to injury symptoms in the form of chlorosis (reduction in the chlorophyll content). The reduction in chlorophyll content after foliar application of fenoxaprop-p-ethyl is likely to be due to its incorporation into the cell membrane function through physiological processes, such as depolarization of membrane potential [20] which is not clarified yet. Subsequently, this makes the translocation of fenoxaprop-p-ethyl become more difficult and thus, relatively large amount of the herbicide retained in the treated leaf tissue and reduce the chlorophyll content [21]. Moreover, this reduction may be due to the enhanced activity of chlorophyll degrading enzyme chlorophyllase and/or disruption of the fine structure of chloroplast and instability of chloroplast or pigment-protein complex, which leads to oxidation of chlorophyll and decreased its concentration.

The results also showed that the reduction in chlorophyll content in resistant biotype treated with fenoxaprop-p-ethyl was lower than that of susceptible biotype of *E. crus-galli*. Furthermore, the results indicated that the chlorophyll content of the treated resistant biotype of *E. crus-galli* again increased more than the untreated one after 21 days of fenoxaprop-p-ethyl application. The possible mechanism of lower reduction or reincrease of chlorophyll content in the resistant biotype of* E. crus-galli* relative to the susceptible one, may be due to the relatively faster metabolism of fenoxaprop-p-ethyl through glycosylation [22] which occurs relatively high in the resistant biotype. That is, relatively small amount of the compound was retained in the treated leaf tissue and, thus, photosynthesis in the treated leaf was able to occur continuously [21]. The accumulation of carbohydrates that has been found in the leaves treated with ACCase inhibitors [23, 24] support, this point of view. Therefore, even if the plant is treated with a rate close to the lethal dose, the treated plants are still alive and likely to regrow when the phytotoxic compound is degraded below the physiologically active concentration [21].

In this study, there were anatomical differences between resistant and susceptible biotypes of *E. crus-galli* treated with fenoxaprop-p-ethyl with respect to leaf lamina thickness and xylem vessel diameter. Moreover, lamina thickness of treated RBT was increased up to untreated RBT. Moreover, no differences in xylem vessel diameter were found between treated and untreated RBT of *E. crus-galli*. Reduction in leaf lamina thickness in sensitive biotype of* E. crus-galli* treated with fenoxaprop-p-ethyl was a reflection of the decrease in mesophyll cells. The decrease in mesophyll cells may be attributed to inhibition of cell division and/or cell enlargement which subsequently may be due to the disruption in plasma membrane that mainly consists of phospholipids. Therefore, any reduction in fatty acids biosynthesis due to fenoxaprop-p-ethyl application that is known as fatty acids synthesis inhibitor [25–27] will affect the membrane formation and subsequently its functions such as cell division and/or cell enlargement. The inhibition in cell division and/or ell enlargement resulted in reduction in mesophyll cells and subsequently in leaf laminal thickness. 

From all previous data, fatty acids are critical components of cell membranes, therefore, reduction or inhibition of fatty acids biosynthesis will affect chlorophyll content and plasma membrane functions. Since that fenoxaprop-p-ethyl is known as fatty acids biosynthesis inhibitor [25–28], its foliar application lead to reduction in chlorophyll content and disruption in plasma membrane functions and subsequently the photosynthesis, number of mesophyll cells, and growth indicators such as growth reduction fifty. All of these parameters were recorded in this study for the treated sensitive biotype of *E. crus-galli* with fenoxaprop-p-ethyl. However, for the resistant biotype of *E. crus-galli* a slight reduction was recorded relative to the sensitive one and this may be due to the fact that the reduction in fatty acids biosynthesis was much lower than that of sensitive biotype.

Therefore, the resistance mechanism of *E. crus-galli* to the fenoxaprop-p-ethyl may be conferred by two proposed mechanisms. Firstly, the mechanism may be due to an alteration in the gene(s) of target site enzyme (ACCase) protein likely the mechanism that confers resistance of *E. crus-galli* to the fenoxaprop-p-ethyl herbicide. The alteration or changes in the protein of ACCase enzyme (the target of fenoxaprop-p-ethyl) in the resistant biotype compared to the susceptible one, induced low affinity of fenoxaprop-p-ethyl herbicide to bind with the target enzyme and the enzyme became insensitive to the herbicide. Moreover, it is apparent that the altered sensitivity of ACCase of the resistant *Echinochloa* sp. to fenoxaprop-p-ethyl may be due to a mutation of the target site enzyme which does not affect equally the bending of the other aryloxyphenoxypropionate [26]. Similarly, the relatively high growth reduction dose of the resistant* E. crus-galli *biotype in this study to the herbicide, chlorophyll content and anatomical differences of the two* E. crus-galli* biotypes provides additional support to this proposed mechanism of resistance.

The second mechanism of *E. crus-galli* resistant to fenoxaprop-p-ethyl may be due to the relatively faster metabolism of fenoxaprop-p-ethyl through glycosylation [22] occurs relatively high in the resistant biotype. That is which relatively small amount of the compound was retained in the treated leaf tissue and, thus, photosynthesis in the treated leaf was able to occur continuously [21]. There are some reports that revealed that an accumulation of carbohydrates has been found in the leaves treated with ACCase inhibitors [23, 24]. Therefore, even if the plant is treated with a rate close to the lethal dose, the treated plants are still alive and likely to re-grow when the phytotoxic compound is degraded below the physiologically active concentration which may be due to the faster metabolism of fenoxaprop-p-ethyl. This proposed mechanism was in agreement with chlorophyll data in this study. Both proposed mechanisms to fenoxaprop-p-ethyl and other ACCase inhibitors had been reported before for another weed [28]; however, against *E. crus-galli* based on physioanatomical differences, this is considered to be the first report.

## 5. Conclusions

There were significant differences between susceptible and resistant biotypes of *E. crus-galli* treated with fenoxaprop-p-ethyl with respect to chlorophyll content, growth reduction, and anatomical test. These differences concluded the ability to identify the occurrence of *E. crus-galli* resistant to fenoxaprop-p-ethyl and assumed that the resistance mechanism was explained either by target site insensitivity or by an enhanced rate of metabolism.

## Figures and Tables

**Table 1:** Effect of fenoxaprop-p-ethyl on the susceptible and resistant biotypes of *E. crus-galli* expressed as the rates of the herbicide required for 50% reduction of the aboveground biomass (GR_50_) and estimated resistance ratio.

Weed biotype	GR_50_ gm a.i/ha	*b*	*c*	*d*	*R^2^*	R/S value	*P* value
Susceptible	3	1.47	0.72	97	0.99	—	<0.05
Resistant	36.21	1.79	1.35	100	0.99	12.07	<0.05

*c*: the mean response (fresh weight as percent of control) at very high herbicide rate.

*d*: the mean response (fresh weight as percent of control) at zero herbicide rate.

*b*: slope of the line.

GR_50_: herbicide rate to reduce plant growth by 50% relative to untreated control.

*R^2^*: the coefficient of determination.

R/S ratio: the GR_50_ of the resistant biotype divided by the GR_50_ of the susceptible biotype.

*P* value: the probability of the obtained results.

**Table 2:** Chlorophyll contents in susceptible and resistant biotypes of* E. crus-galli* after 14 days of treatment with fenoxaprop-p-ethyl compared with untreated ones.

Treatments	Chlorophyll pigments (mg/L)		
	A	B	Total
Susceptible (control)	3.001^a^	1.785^a^	5.211^a^
Susceptible + F	1.872^c^	0.552^d^	2.425^d^
Resistance (control)	2.494^b^	1.461^b^	3.956^b^
Resistance + F	1.458^d^	1.175^c^	2.633^c^

*F = fenoxaprop-p-ethyl.

^a,b,c,d^indicate the significance and non-significance between means using Duncan multiple range test.

**Table 3:** Chlorophyll contents in resistant untreated and resistant treated biotypes of* E. crus-galli* after 21 days of treatment.

Treatments	Chlorophyll pigments (mg/L)		
	A	B	Total
Resistance (control)	3.126^b^	1.927^b^	5.011^b^
Resistance + F	3.320^a^	2.083^a^	5.403^a^

*F = fenoxaprop-p-ethyl.

^a,b^indicate the significance and non-significance between means using Duncan multiple range test.

**Table 4:** Some anatomical parameters in the two sensitive and resistant biotypes of *E. crus-galli*, that is, laminal thickness and vessel diameters after 14 days of treatment with fenoxaprop-p-ethyl compared with untreated ones.

Treatments	Laminal thickness (*μ*m)	Xylem vessels diameter (*μ*m)
Susceptible (control)	130^c^	35^a^
Susceptible + F	34^a^	17^c^
Resistance (control)	137^c^	36^a^
Resistance + F	51^b^	25^b^

*F = fenoxaprop-p-ethyl.

^a,b,c^indicate the significance and non-significance between means using Duncan multiple range test.

**Table 5:** Some anatomical parameters in recovered treated resistant biotypes of *E. crus-galli*, that is, laminal thickness and vessel diameters after 21 days of treatment with fenoxaprop-p-ethyl compared with untreated resistant one.

Treatments	Laminal thickness (*μ*m)	Vessels diameter (*μ*m)
Resistance (control)	138^a^	34^b^
Resistance + F	155^b^	34^a^

*F = fenoxaprop-p-ethyl.

^a,b^indicate the significance and non-significance between means using Duncan multiple range test.
